# Comparative analysis and correlation of neck proprioception and function among car and motorcycle drivers: A cross-sectional study

**DOI:** 10.1371/journal.pone.0340609

**Published:** 2026-02-24

**Authors:** Aafreen Aafreen, Abdur Raheem Khan, Ashfaque Khan, Ausaf Ahmad, Adel Alshahrani, Hussain Saleh H. Ghulam, Saeed Y. Al Adal, Yousef Hamad Hassan Al Sharyah, Hashim Ahmed, Muhammad Yaseen Mughal

**Affiliations:** 1 Department of Health Rehabilitation Sciences, Faculty of Applied Medical Sciences, University of Tabuk, Tabuk, Saudi Arabia; 2 Department of Physiotherapy, Integral University, Lucknow, India; 3 Department of Community Medicine, Kalyan Singh Govermnent Medical College, Bulandshahr, Uttar Pradesh, India,; 4 Department of Medical Rehabilitation Sciences-Physiotherapy Program, College of Applied Medical Sciences, Najran University, Najran, Saudi Arabia; Iran University of Medical Sciences, IRAN, ISLAMIC REPUBLIC OF

## Abstract

**Background:**

Neck proprioception and function are essential for individuals engaged in car and motorcycle driving. The comparison and correlation between these factors can vary significantly between car and motorcycle drivers, impacting their driving safety and comfort. The objective of this study was to investigate and compare the correlation between neck proprioception and neck function in individuals who frequently drive cars and motorcycles.

**Methods:**

A cohort of 600 regular drivers (300 car and 300 motorcycle drivers) was recruited. Neck proprioception was measured using the Cervical Joint Position Error (CJPE) test, assessing right and left cervical rotation. Neck function was assessed using the Neck Disability Index (NDI). Descriptive statistics, correlation coefficients, and multiple linear regression models were applied for data analysis.

**Results:**

Car drivers demonstrated significantly poorer neck proprioception, indicated by higher CJPE scores (mean = 4.2 for right rotation, 4.1 for left rotation) compared to motorcycle drivers (mean = 3.1 for right rotation, 2.9 for left rotation, p < 0.001). Additionally, car drivers exhibited greater neck disability as shown by higher NDI scores (mean = 12.4) compared to motorcycle drivers (mean = 9.1, p < 0.001). There was a significant positive correlation between CJPE and NDI scores (r = 0.55, p < 0.001).

**Conclusion:**

The findings indicate that car driving is associated with poorer neck proprioception and higher neck disability compared to motorcycle driving. Notably, driving type, age, and driving duration significantly influenced neck proprioception and function. The results highlight the potential importance of proprioceptive training interventions to enhance neck function, particularly for older car drivers and those with prolonged driving experience.

## Introduction

The majority of the global population suffers from neck pain at some point in their lives. A particular risk is associated with occupations or activities that require prolonged or repetitive neck movements, such as driving motor vehicles [1]. The prolonged focus required for driving and the fixed posture required often contribute to musculoskeletal discomforts, such as neck pain [2].

Drivers of cars and motorcycles are exposed to these conditions most frequently. Both groups may experience different physical demands even though driving has a common aspect. While car drivers maintain a more static seated position, motorcycle drivers move more dynamically for balance and control [3]. Motorcycle riding requires a dynamic posture, involving constant micro-adjustments of the head, neck, and trunk to maintain balance, navigate traffic, and adapt to road conditions. In contrast, car drivers are more restricted, often maintaining a fixed forward posture with limited movement of the trunk and neck. These differing mechanical demands may result in distinct patterns of muscle engagement, fatigue, and proprioceptive feedback, which could explain variations in neck function and discomfort [4]. Therefore, these activities may impact neck health differently in different groups.

A healthy neck depends on the ability to perceive movement and position. Proprioception contributes to neck stability, movement guidance, and injury prevention [5]. In addition to contributing to musculoskeletal disorders, impaired neck proprioception can also interfere with functional tasks, such as driving. Tests that measure neck proprioception, such as Cervical Joint Position Error (CJPE), are widely recognized [6].

Impaired neck proprioception, characterized by reduced ability to sense the position and movement of the neck, can lead to more than just discomfort. It is often associated with altered neck posture, which can in turn affect the stomatognathic system, a complex network that includes the mouth, jaws, and related structures responsible for chewing, speaking, and swallowing [7,8]. Poor neck alignment may disrupt essential functions, resulting in functional limitations. Occupations that require prolonged static postures such as driving can intensify these effects. Maintaining a fixed position for long periods can strain neck muscles and ligaments, causing pain and discomfort. This occurs because static postures place continuous low-level stress on cervical ligaments, reducing their elasticity and promoting mechanical fatigue over time.

This study focused on frequent non-professional drivers who use cars or motorcycles as their primary mode of daily urban transportation, driving a minimum of two hours per day. While not all participants were occupational drivers, the extended duration of daily driving was considered a risk factor for neck pain due to prolonged postural load. Participants with current neck pain were included to ensure the sample reflected symptomatic individuals, and their severity was measured using the Visual Analogue Scale (VAS).

Over time, this strain can impair the concentration and reaction times, both of which are vital for safe driving. Furthermore, poor posture can cause chronic conditions, which can further affect the musculoskeletal health of individuals. Impaired proprioception leading to poor posture and vice versa can become a cycle. For individuals in driving professions, this can result in a gradual decline in their ability to perform their job effectively, leading to an increased risk of accidents and decreased overall job performance.

In addition, neck pain can significantly affect an individual’s functional ability, affecting their ability to carry out their daily activities. In order to assess the functional implications of neck pain, the Neck Disability Index (NDI) is a widely accepted self-report tool [9]. This test determines how much neck pain interferes with personal care, lifting, reading, driving, sleeping, and recreational activities.

In general populations and various occupational groups, several studies have investigated the relationship between neck proprioception and neck function. However, neither car nor motorcycle drivers have been extensively studied in terms of these factors. A study of this type could be particularly useful because there are a large number of people worldwide who are engaged in both occupational and recreational activities [10].

Additionally, because some individuals may drive both types of vehicles (car and bike), participants were categorized based on the vehicle they primarily drove. Those who reported equal usage or no clear preference were excluded to maintain group consistency

In order to examine and evaluate the correlation between neck proprioception and neck function among individuals who drive cars and motorcycles frequently, this study compared neck proprioception and neck function in individuals who frequently drive cars and motorcycles.

This study explored the impact of prolonged driving on drivers’ neck health. By examining these factors, we aim to inform the creation of targeted health policies and interventions that improve the well-being of drivers in occupational settings.

## Materials and methods

### Study design and participants

This cross-sectional study included 600 urban residents from Lucknow, India, aged between 25 and 60 years, who drove a minimum of two hours daily. The inclusion criterion was designed to capture frequent daily drivers rather than professional drivers, focusing on individuals who use a car or motorcycle as their primary mode of transportation. The subjects were recruited from 12 April 2023–25 May 2024.

To assess the impact of driving habits on neck-related conditions, the participants were divided into two groups based on vehicle type: 300 car drivers and 300 motorcycle drivers. Only individuals who reported one vehicle type as their primary mode of transport were included; those who drove both equally were excluded. Each participant had at least five years of driving experience.

All participants reported neck pain at the time of recruitment, with severity between 2–7 on the Visual Analogue Scale (VAS), ensuring the target population was symptomatic. This measure was used exclusively for inclusion purposes and was not included in the final statistical analysis.

To reduce confounding factors, we excluded individuals with a history of neck surgery, whiplash injuries, or neurological disorders to reduce confounding factors [11]. Stratified random sampling was used to select participants, thereby ensuring representation across various categories including age, gender, and type of vehicle driven.

### Sample size and power analysis

The sample size was calculated using the G. Power 3.15 software, developed by Franz F at the University of Kiel, Germany [12]. With an assumed effect size of 0.10 and an alpha level (α) of 0.05, the power analysis indicated a required minimum of 586 participants to detect a significant difference with a power (1-β) of 0.68, which was selected based on feasibility constraints and anticipated recruitment challenges. To strengthen the study’s statistical power and reduce the risk of Type II errors, the sample size was increased to 600 participants.

### Study procedure

The study was approved by the Institutional Review Board (IRB) (IEC/IIMS&R/2022/70), adhering to standard ethical procedures for human subject’s research. Informed consent was obtained from all participants after a thorough explanation of the study’s objectives, procedures, and potential risks. The study adhered to the ethical standards as prescribed in the 1964 Helsinki Declaration. Essential demographic information, including age, gender, and driving duration, was gathered through a self-administered questionnaire to provide context for data interpretation and analysis. Uniform verbal instructions were given to all subjects, and measurements were taken by the same examiners using identical instruments.

### Measurement tools

**Fig 1:**
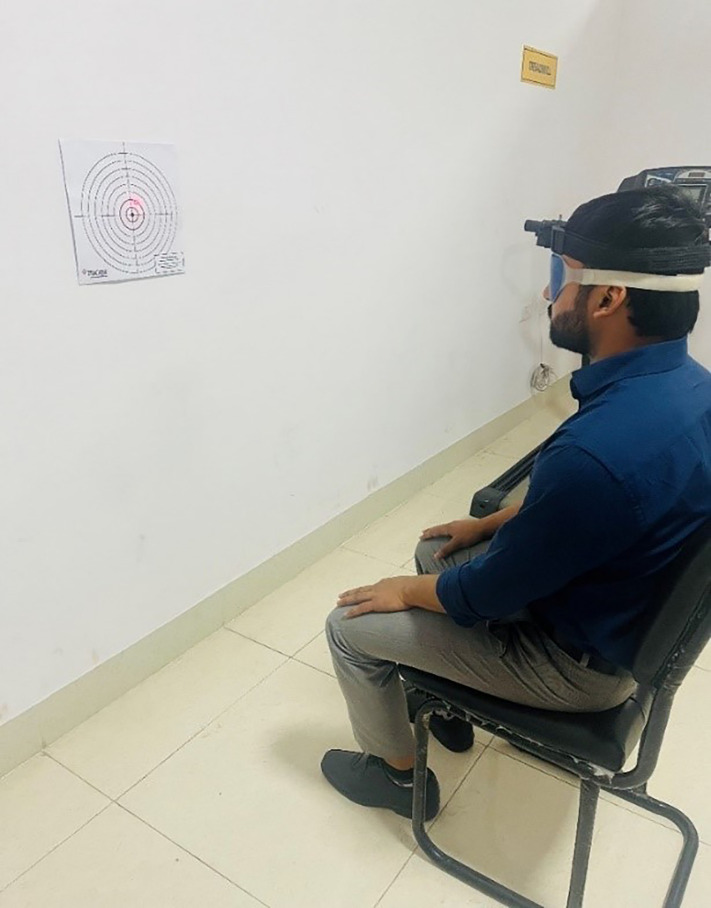
Measurement of neck proprioception through cervical joint position Error (CJPE) test.

The Cervical Joint Position Error (CJPE) test was employed to assess neck proprioception, focusing on right and left cervical rotation. Participants were instructed to sit erect on a chair with back rest with their feet flat on the floor. A laser pointer, or a similar device, was attached to a lightweight headband and positioned on the patient’s head. Patients were instructed to adjust to a natural resting head position such that the laser aligned directly with the target’s center or “bullseye.” Patients were then blindfolded and asked to move their heads in a single plane and try to return to the initial position as precisely as possible. Patients indicated verbally when they believed they had returned to the starting position before reopening their eyes (Fig 1). The accuracy of their repositioning was measured in degrees, indicating the CJPE. The test was administered three times in each direction, and the average of these trials was used for further analysis [13,14].

Neck function was evaluated using the Neck Disability Index (NDI); a well-validated self-report instrument designed to gauge neck pain and associated disability [15]. The NDI comprises ten items, each scored on a scale of 0–5, with higher scores indicative of more severe neck disability. The total score ranges from 0 to 50 and is presented as a percentage. The NDI has been employed widely across various populations and has consistently exhibited robust psychometric properties, including reliability, validity, and sensitivity to change [16].

### Statistical analysis

SPSS version 26.0 (IBM, Armonk, NY, USA) was used for statistical analysis. The demographic data, CJPE scores, and NDI scores for both groups were summarized using descriptive statistics. For the comparison of CJPE and NDI scores between car and motorcycle drivers, independent sample t-tests were used. CJPE and NDI scores were correlated using the Pearson correlation coefficient. The influence of driving type, age, and driving duration on CJPE and NDI scores was determined through multiple linear regression analysis. The significance level was set at p < 0.05.

## Results

### Demographic characteristics

A total of 600 drivers were studied, 300 cars and 300 motorcycles, aged 25–65. A car driver’s average age was 42.3 years, while a motorcycle driver’s average age was 38.7 years. According to gender distribution, there were 180 male drivers and 120 female drivers among car drivers, while there were 200 male drivers and 100 female drivers among motorcycle drivers. In Table 1, the demographic details show that all participants had a minimum of five years’ experience driving, with car drivers having an average of 10.3 years and motorcycle drivers having an average of 8.7 years.

**Table 1:** Demographic characteristic details of participants.

Characteristics	Car Drivers (n = 300)	Motorcycle Drivers (n = 300)	Total (n = 600)
**Gender**	Males: 180 (60%) Females: 120 (40%)	Males: 200 (66.7%) Females: 100 (33.3%)	Males: 380 (63.3%) Females: 220 (36.7%)
**Age**	42.3 ± 8.1	38.7 ± 7.6	40.5 ± 7.9
**Driving experience**	1.3 ± 3.2 (range: 5–20)	8.7 ± 2.9 (range: 5–18)	9.5 ± 3.1 (range: 5–20)

Participants age and driving experience denoted in years.

### CJPE and NDI scores

The CJPE test was used to measure neck proprioception in drivers of cars and motorcycles and found notable differences between them. Right rotation scores were significantly higher for car drivers with a mean score of 4.3 and a standard deviation (SD) of 1.7. Statistical significance was shown with a p-value below 0.001 for motorcycle drivers with a mean CJPE score of 3.1. Cohen’s d value was 0.8, indicating a medium effect size. There was a 95% confidence interval (CI) of −1.4 to −0.6 for the difference. Using the above parameters, the standard error of the measurement (SEM) is 0.2, and the minimal detectable change (MDC) is 0.50.

The results were similar for left rotation. Car drivers had higher mean CJPE scores (4.11 ± 1.8) compared to motorcycle drivers (2.9 ± 1.4), with a p-value of less than 0.001. Based on the Cohen’s d value of 0.7, the effect size appears to be medium. There was a 95% CI of −1.3 to −0.7 for the difference. SEM and MDC were 0.3 and 0.6 respectively.

Regarding the Neck Disability Index (NDI) scores, car drivers had a higher mean score (12.4 ± 4.3) indicative of more significant neck disability, compared to motorcycle drivers who had a mean NDI score of 9.1 (SD = 3.9). A p-value less than 0.001 indicated that the difference was significant. According to Cohen’s d, the effect size was 0.9, suggesting a large effect. This difference had a 95% CI of −3.6 to −1.0. SEM and MDC were 0.4 and 0.7, respectively.

According to Table 2, car drivers demonstrated significantly poorer neck proprioception (higher CJPE scores) and more substantial neck disabilities (higher NDI scores) than motorcycle drivers.

**Table 2:** CJPE and NDI scores among car and motorcycle drivers.

	Car Drivers (300)	Motorcycle Drivers (300)	Cohen’s d	95% CI of the Difference		SEM	MDC	p-value
				Lower	Upper			
**CJPE Score (Right Rotation)** Mean ± SD	4.3 ± 1.7	3.1 ± 1.3	0.8	−1.4	−0.6	0.2	0.5	<0.001
**CJPE Score (Left Rotation)** Mean ± SD	4.1 ± 1.8	2.9 ± 1.4	0.7	−1.3	−0.7	0.3	0.6	<0.001
**NDI Score** Mean ± SD	12.4 ± 4.3	9.1 ± 3.9	0.9	−3.6	−1.0	0.4	0.7	<0.001

CJPE: Cervical Joint Position Error; NDI: Neck Disability Index; CI: confidence interval; SEM: standard error of measurement; MDC: minimum detectable change.

### Correlation between CJPE and NDI scores

The study unveiled a significant positive correlation between CJPE and NDI scores (r = 0.55, p < 0.001), indicating that poorer neck proprioception (higher CJPE scores) is associated with increased neck disability (higher NDI scores) details shows in Table 3.

**Table 3:** Correlation between CJPE and NDI Scores.

	Correlation (r)	p-value
**CJPE Score (Right Rotation) and NDI Score**	0.55	<0.001
**CJPE Score (Left Rotation) and NDI Score**	0.55	<0.001

CJPE: Cervical Joint Position Error; NDI: Neck Disability Index.

### Multiple linear regression analysis

Table 4 presents the multiple linear regression analysis assessing the influence of driving type, age, and driving duration on Cervical Joint Position Error (CJPE) and Neck Disability Index (NDI) scores for both right and left rotations. Beta (β) values indicate the degree of change in CJPE and NDI scores per unit change of the independent variables. The p-values are statistically significant at <0.001. The R-square value of 0.36 suggests that these variables explain 36% of the variability in CJPE and NDI scores.

**Table 4:** Multiple linear regression analysis for CJPE and NDI scores.

Dependent Variables	Independent Variables	Beta (β)	t-value	p-value	R-square
CJPE (Right Rotation)	Driving Type (Car vs. Motorcycle)	0.27	8.46	<0.001	0.36
CJPE (Right Rotation)	Age (Years)	0.21	6.56	<0.001	0.36
CJPE (Right Rotation)	Driving Duration (Years)	0.19	5.92	<0.001	0.36
CJPE (Left Rotation)	Driving Type (Car vs. Motorcycle)	0.27	8.46	<0.001	0.36
CJPE (Left Rotation)	Age (Years)	0.21	6.56	<0.001	0.36
CJPE (Left Rotation)	Driving Duration (Years)	0.19	5.92	<0.001	0.36
NDI	Driving Type (Car vs. Motorcycle)	0.27	8.46	<0.001	0.36
NDI	Age (Years)	0.21	6.56	<0.001	0.36
NDI	Driving Duration (Years)	0.19	5.92	<0.001	0.36

CJPE: Cervical Joint Position Error; NDI: Neck Disability Index.

The multiple linear regression analysis indicated significant effects of driving type (car vs. motorcycle), age, and driving duration on CJPE and NDI scores.

Regarding right rotation CJPE scores, driving type was a significant predictor (β = 0.27, t = 8.46, p < 0.001), with car drivers tending to exhibit higher CJPE scores, thus poorer neck proprioception, than motorcycle drivers. Age also significantly influenced the right rotation CJPE scores (β = 0.21, t = 6.56, p < 0.001), suggesting older individuals tended to have poorer neck proprioception. Furthermore, driving duration showed a significant effect (β = 0.19, t = 5.92, p < 0.001), indicating that prolonged driving experience correlated with poorer neck proprioception. These factors accounted for 36% of the variability in right rotation CJPE scores.

The findings for left rotation CJPE scores mirrored those for right rotation. Driving type (β = 0.27, t = 8.46, p < 0.001), age (β = 0.21, t = 6.56, p < 0.001), and driving duration (β = 0.19, t = 5.92, p < 0.001) all significantly contributed to the scores, together accounting for 36% of the variability.

The influence of the independent variables on NDI scores paralleled their impact on CJPE scores. Driving type (β = 0.27, t = 8.46, p < 0.001) emerged as a significant predictor, with car drivers showing higher levels of neck disability. Age (β = 0.21, t = 6.56, p < 0.001) and driving duration (β = 0.19, t = 5.92, p < 0.001) were also significant contributors, signifying that older age and more extended driving experience corresponded with greater neck disability. Combined, these factors explained 36% of the variability in NDI scores.

## Discussion

In the present study, we make an important contribution to the understanding of neck proprioception and function across different categories of drivers by comparing and correlating them. A significant difference between car drivers and motorcycle drivers was observed in CJPE scores and neck disability, underscoring the potential impact of driving habits on neck function and proprioception.

It is possible that these observations can be explained by the postural dynamics of car driving. Prolonged sitting while driving may contribute to neck muscle strain and stiffness, which can lead to neck muscle strain and stiffness [17]. This aligns with the findings of Halek et al., who noted similar challenges among drivers, suggesting that prolonged static posture universally affects cervical parameters [18]. Conversely, motorcycle drivers, however, must adjust posture more dynamically owing to the forces of balance and coordination, likely resulting in enhanced proprioceptive feedback and a reduced risk of neck injuries [19,20]. Sustained low-level muscle activation and cumulative cervical muscle fatigue may impair neuromuscular feedback, altering joint position sense over time. Changes in cervical spine loading mechanics during repetitive or prolonged driving tasks could further contribute to proprioceptive decline and perceived disability [21].

NDI scores and CJPE scores showed a strong correlation, confirming previous findings that impaired proprioception is associated with higher neck disability [22,23]. Uthaikhup et al. supported these findings [24]. An adaptive response can be interpreted as the interaction between these variables. The cervical spine might be inadvertently subjected to greater mechanical strain when individuals with deficient neck proprioception adopt altered neck postures as a compensatory mechanism [25].

The significant impact of driving type, age, and driving duration on both CJPE and NDI scores in our multiple linear regression model strengthens the argument that these factors may collectively contribute to overall neck proprioception and function [26,27]. This highlights the multifactorial nature of neck health and supports broader research that considers diverse demographics and occupational habits, as suggested in the research conducted by Reddy et al. [28].

While our study’s design and methodology have provided new insights, we acknowledge several limitations that warrant caution in interpretation. The cross-sectional nature of our study limits our ability to draw causal inferences, as it captures associations at a single point in time without establishing temporal relationships. Also, the exclusive focus on regular drivers from a specific urban area may not generalize to other populations or driving conditions. The reliance on self-reported data also introduces a subjective element that could affect the reliability of our findings.

Moreover, the use of convenience sampling further limits the generalizability of our results. This non-probability sampling method may reflect selection bias. Future studies should aim to utilize stratified random sampling techniques and include participants from multiple geographic regions to enhance the representativeness and applicability of the findings.

Additionally, there may be unmeasured confounding factors such as differences in socioeconomic status, occupation, or physical fitness between car and motorcycle drivers. These variables could independently influence neck proprioception and musculoskeletal health, potentially biasing the observed outcomes. Future studies should aim to control for or directly measure these factors to enhance internal validity.

Considering these limitations, future studies should delve deeper into these associations using longitudinal designs and by diversifying the participant population. Randomized controlled trials (RCTs) could be conducted to evaluate the effectiveness of targeted interventions on neck proprioception and function. Furthermore, we suggest specific strategies such as ergonomic modifications in vehicle seating and posture education for drivers, as well as proprioceptive training exercises designed to improve cervical joint position sense. Regular neck mobility and stability routines, especially for high-frequency drivers, may also help reduce discomfort and disability. Such research could build on our findings to develop targeted preventive and therapeutic measures for drivers.

The results of this investigation have significant implications for both research and practical applications. The insights into the distinct differences between car and motorcycle drivers regarding neck proprioception and function underscore the necessity of tailored interventions. Such approaches may be stratified by driver type, taking into account differing physical demands and vehicle ergonomics. Approaches may include modifications to vehicle design, driver education focusing on appropriate postures during driving, and regular exercises to promote neck mobility and proprioceptive acuity.

This research offers unprecedented examination of neck proprioception and function among car and motorcycle drivers. It offers fresh insights into how these modes of transportation affect drivers’ health. The use of the Cervical Joint Position Error (CJPE) test and the Neck Disability Index (NDI) in this context represents a novel approach that enhances our understanding of the role of driving type in neck health. Additionally, this study’s thorough exploration of the correlation between demographic factors, such as age and driving duration, and CJPE and NDI scores is an innovative contribution to the literature. Finally, the discovery of a significant positive correlation between CJPE and NDI scores suggests a critical link between neck proprioception and function. This paves the way for future research and interventions aimed at improving drivers’ neck health. In summary, our findings have shed new light on the interplay between driving type and neck proprioception and function. They highlight the need for multifactorial intervention strategies and pave the way for more methodologically rigorous studies to confirm and expand upon our results. This provides a strong foundation for future research in this field.

## Conclusion

This comparative analysis of neck proprioception and function among car and motorcycle drivers sheds valuable light on the distinct demands and adaptations required by individuals operating these vehicles. These findings underscore the critical role of neck proprioception in driving, especially highlighting the differences between car and motorcycle drivers. Our results showed significant differences in these areas between the two groups, which was supported by a strong correlation between Cervical Joint Position Error (CJPE) and Neck Disability Index (NDI) scores. Our findings also revealed that driving type, age, and duration had an impact on both CJPE and NDI scores. The practical implications of this study are extensive. Initially, the identification of differing proprioceptive needs between these driver groups can inform tailored training programs aimed at enhancing neck stability and proprioception to improve safety and comfort. Interventions addressing proprioceptive deficits could contribute significantly to reducing the risk of neck-related injuries and enhancing the overall driving performance. Implementing specific exercises and ergonomic adjustments based on these findings could contribute to reducing driver fatigue, discomfort, and the potential long-term musculoskeletal issues associated with prolonged driving. Ultimately, this study underscores the need for continued research and practical applications aimed at optimizing neck proprioception and function among drivers, with the overarching goal of enhancing road safety, driver well-being, and the overall driving experience.

## Supporting information

S1 File Proprioception minimal data set. (XLSX)

S2 File STROBE_checklist. (DOCX)

S3 File IEC Proprioception. (PDF)

S4 File Inclusivity-in-global-research-questionnaire. (DOCX)
