# Supplementary material for: Comparative analysis and correlation of neck proprioception and function among car and motorcycle drivers: A cross-sectional study
Source: PLoS One. 2026 Feb 24;21(2):e0340609. doi: 10.1371/journal.pone.0340609 (PMC12931761; doi:10.1371/journal.pone.0340609)
Supplement: S3 File — (PDF) [file pone.0340609.s003.pdf]

# INSTITUTIONAL ETHICS COMMITTEE (IEC)

## IIMS&R INTEGRAL UNIVERSITY, LUCKNOW

IEC/IIMS&R/2022/ 40

# CERTIFICATE

This is to certify that research work entitled **"Impact of car and bike driving on cervical range of motion, craniovertebral angle and proprioception among individuals with and without neck pain"** submitted by **Dr. Abdur Raheem Khan, Aafreen** for ethical approval before the Institutional Ethics Committee IIMS&R.

The above mentioned research work has been approved by Institutional Ethics Committee, IIMS&R with consensus in the meeting held on **19 May 2022**.

**Dr.Deepak Chopra**
(Jt.Member Secretary)
IRC/IEC
IIMS &R

**Dr.Q.S.Ahmed**
(Member Secretary)
IRC/IEC
IIMS &R
